# Abstracts and Gleanings

**Published:** 1884-10-20

**Authors:** 


### ABSTRACTS AND GLEANINGS.

**Vaccine Virus Efficacious Without the Production of Cutaneous Manifestations**—(*Rev. mens. des Mal. de l' E.*): The usual supposition is that vaccination has been ineffective if, after several days, the characteristic pustules do not appear. That this is not always the case appears from the experience of the author of this paper. He vaccinated, upon the same day, five infants from three to five months of age with virus from a young heifer, obtained fresh a few hours before it was used. In two cases the vaccination took unmistakably. In a third, after a few days, there was swelling and also pain at the point of vaccination puncture—the operation having been performed by thrusting a cannulated needle, charged with the virus, into the muscular tissue of the arm. An abscess was supposed to be imminent, and treatment proper for such a condition was adopted, but no abscess resulted. In the fourth case swelling was also noticed at the points of puncture ten days after the operation. In the fifth case there was also slight local swelling. After ten days there remained of the swelling, in these three cases (which alone are of interest in this connection), nothing but a few small nodosities, which, however, could be plainly felt, either in the fatty or the muscular tissue. Traces of them remained for more than six weeks. While they were yet plainly perceptible, revaccination was practiced, with all possible precautions, in both arms in each case. Results: In one, redness and swelling in thirty-six hours in both arms, an evidence of the so-called false vaccination, which is a local manifestation that the patient is still protected by an antecedent vaccination. In the other two cases no result was perceptible. A third vaccination was performed upon one of them, false vaccination resulting. These facts, together with the circumstance that similar ones have been observed in the inoculation of sheep, lead to the belief that the vaccinal pustule is not a necessary consequence in order to effectual vaccination; in other words, that some people can acquire the protection afforded by vaccine without the customary eruption. It is also undoubtedly true that there are people who are especially rebellious to the influence of vaccine, and still others with whom the period of incubation may be protracted through two, three, or even four weeks, but these statements do not militate against the former ones, which are substantiated by that most convincing agency, personal experience.—*Archives of Pediatrics.*

**On Opening and Drainage of Abscess Cavities in the Brain.**—The antiseptic method of operating and after-treatment has not, as yet, been fully tested in operations upon the brain. This is natural, for not only have we inherited a just dread of dealing with an organ, the large majority of whose diseases are dangerous or fatal, but our knowledge of the physiological functions of the brain and of their pathological modifications being extremely limited, we are not in a position to form such an accurate diagnosis as calls for surgical interference. Drs. Christian Fenger and E. W. Lee, of Chicago, in an extremely interesting paper on this subject in the July number of The American Journal of the Medical Sciences, consider the treatment of traumatic cerebral abscess, and report a case which was successfully treated by opening and drainage.

Bergman, in discussing the treatment of cerebral abscess, unhesitatingly sets it down as an axiom that wherever there is an accumulation of pus, trephining is most clearly and indubitably indicated, for the opening of an abscess in the brain is as necessary as in any other part of the body, and, we would add, even more so. A correct diagnosis of abscess having been made, the further difficulty presents itself of locating it with sufficient accuracy, so as to be able to find it. A number of cases are on record in which a correct diagnosis had been made, the trephine also put on more or less at the right place, and the knife or trocar being passed into the brain, nevertheless missed the abscess. Drs. Fenger and Lee show by their case that this difficulty can be obviated by multiple exploratory aspirations, performed at interstices sufficiently small to prevent any abscess from escaping detection, even if the trephine opening should not have been made at the point of the skull nearest the abscess.

There are on record a large number of cases of cerebral abscess in which trephining was performed, pus evacuated and temporary relief obtained; but later relapse followed and a fatal termination ensued. It is possible, judging from the success the practice has met with in the treatment of abscesses in other situations, that drainage of the cerebral abscess-cavity, with or without washing out, would have saved some of these cases by preventing the re-accumulation of pus and the continuous infection of the surrounding brain tissue, the acute œdema of which is well known to be, as a rule, the final cause of death. As far as Drs. Fenger and Lee are aware, draining and washing out of cerebral abscess-cavities has heretofore not been tried; that it can be effected, and without any detriment to the patient, is shown by their case, the treatment of which, they hold, strictly conforms to the rational methods of modern surgery in treating abscesses in general; and because of this, and not because their patient recovered, they regard the case as answering affirmatively the question: Is it probable that abscesses of the brain can be treated advantageously on the same principles as abscesses in other parts of the body?—*Med. Age.*

**The Castration of Women.**—Dr. Wilhelm Tauffer, of Buda-Pesth, gives, in the Zeitschrift fur Geburts hulfe und Gynakologie, an account of twelve cases of this operation performed by himself, and then presents the followiug conclusions (Medical Times):

1. Castration is an operation which, with proper care, is not attended with any great risk, the unavoidable mortality being now less than 10 per cent.

2. The operation should be performed with antiseptic precautions and under carbolic spray; the abdominal cavity should be closed; drainage is only exceptionally required.

3. The limitation that castration is not called for when the climacteric is near can only be conditionally accepted, because the age at which the climacteric occurs is very different in different individuals, and cannot be foretold.

4. The condition laid down by Hezar, that the ovaries should be distinctly felt before their extirpation is attempted, is an impracticable one.

5. Both ovaries should be removed, even if the disease be limited to one; excepting in cases where special circumstances make it desirable to leave behind the apparently healthy ovary. The reason for removing both ovaries is, that the remaining gland has a tendency to become diseased in the same way as its fellow.

6. It is generally desirable to remove the tubes as well as the ovaries; and it is unconditionally necessary if there be the slightest appearance of disease in them.

7. Hystero-epilepsy is curable by castration.

8. The symptoms grouped together under the name of hysteria, when rightly analyzed, can often be traced back to ovarian disease.

9. The question as to the influence upon uterine fibroids of the ligature of large nutrient-vessels going to them, without castration, is worth consideration.

10. With regard to prognosis, it is important to remember that the menopause only follows immediately upon the operation in those cases in which the neighboring organs are not diseased; but all inflammatory conditions of them delay the climacteric.

11. The final result of castration can only be determined after the lapse of months.

12. It must be regarded as an open question how far diseases of the female sexual organs influence the development of certain psychoses.

13. So must the question whether such psychoses are curable by castration.

14. In the interest of unity and comparison of observations, it is desirable that the classification of cases (suitable for this operation) adopted by Hezar should be generally accepted.—*Journal Amer. Med. Assoc'n.*

**Hot Water and Inflamed Mucous Surfaces.**—Dr. George R. Shepard, Hartford, Conn., says (in the Medical Record): "I have used hot water as a gargle for the past six or eight years, having been led to do so from seeing its beneficial effects in gynecology. In acute pharyngitis and tonsillitis, if properly used at the commencement of the attack, it constitutes one of our most effective remedies, being frequently promptly curative. If used later in the disease, or in chronic cases, it is always beneficial, though, perhaps, not so immediately curative. To be of service it should be used in considerable quantity (a half pint or pint) at a time, and just as hot as the throat will tolerate. I have seen many cases of acute disease thus aborted, and can commend the method with great confidence. I believe it may be taken as an established fact that, in the treatment of inflammations generally, and those of the mucous membrane in particular, moist heat is of service, and in most cases hot water is preferable to steam. All are familiar with its use in ophthalmia and conjunctivitis, as also in inflammation of the external and middle ear, and I feel confident that those who employ it for that most annoying of all slight troubles to prescribe for, viz: a cold in the head, or acute coryza, will seldom think of using the irritating drugs mentioned in the books, nor of inducing complete anæsthesia with chloroform in preference to the hot water douche. It is important to recollect, however, that, to be effective in this disease, it must be employed in the first stage of hyperæmia. Nor is the urethral mucous membrane any exception to the general rule. Cases of urethritis and gonorrhœa are benefitted or cured by its use when applied to the commencement of the inflammation. In the cure of gleet, I know of no other agent so often serviceable. Where there is stricture, almost any case of gleet will yield in a short time to the use of the hot water douche followed by mild astringents. Keep in mind the principle of its curative action, viz: the removal of hyperæmia, and the local sedative influence, and there will be found few cases of inflammation of mucous membranes where it will not, at some state of the disease, promote a cure.—*Weekly Med. Review.*

**Glycerine as a Remedy in Indigestion.**—The editor of the Medical Index has found the exhibition of glycerine to be attended with satisfactory results in two forms of indigestion, particulary. First, in that form of irritative dyspepsia which is the common result of rapid eating and imperfect mastication. The usual symptom in such cases is distress coming on half an hour or an hour after meals. There is, also, duodenal catarrh and dyspepsia, with, perhaps, slight jaundice and other symptoms, referable to, and explained by the irritated mucous membrane of the stomach and duodenum. The indications in such cases are well-defined. The food must be prevented from undergoing mischievous chemical changes before it can be acted upon by the enfeebled digestive organs. and a remedy must be given which shall exert a local soothing effect upon the irritated mucous surface. Glycerine, theoretically, from its preserving and emollient properties, fulfills these indications, and in practice our contemporary has not been disappointed in its use.

A somewhat similar condition to the above is met with among children shortly after birth, after a trial of feeding them solid food has been followed by colic and soothing syrup. In such cases the child is apt to have greenish discharges, occasionally specked with blood. Glycerine will be found an admirable remedy in these cases.—*Ther. Gaz.*

**Tyndall's Theory of Explaining the Immunity Obtained against a Second Attack of Contagious Disease.**—Professor Tyndall, in the Pall Mall Gazette, discusses the above subject in the following ingenious manner: "One of the most extraordinary and unaccountable experiences in medicine was the immunity secured by a single attack of a communicable disease against future attacks of the same kind. Smallpox, typhoid or scarlatina, for example, was found, as a general rule, to occur only once in the lifetime of the individual, the successful passage through the disorder apparently rendering the body invulnerable. Reasoning from analogy, I have ventured to express the opinion that the rarity of second attacks of communicable diseases was due to the removal from the system, by the first parasitic crop, of some ingredient necessary to the growth and propagation of the parasite.

The cultivation of micro-organisms, which is now everywhere carried on, enables us to realize the smallness of the changes which, in many cases, suffice to convert a highly nutritious liquid into one incapable of supporting microscopic life. Various important essays bearing upon this subject have been recently published in the Revue Scientifique. M. Bouley there draws attention to the results obtained by M. Raulin in the cultivation of a microscopic plant named aspergillus niger. The omission of potash from Raulin's liquid suffices to make the product fall to 1-25 of the amount collected when potash is present. The addition of an infinitesimal amount of a substance inimical to the life of a plant is attended with still more striking results. For example, one part in 1,600,000 of nitrate of silver added to the liquid entirely stops the growth of the plant. And now we come to the important application of this fact which has been indicated by M. Duclaux. Supposing the aspergillus to be a human parasite—a living contagium—capable of self-multiplication in the human blood, and of so altering the constitution of that liquid as to produce death; then, the introduction into the blood of a man weighing sixty kilograms of five milligrams of the nitrate of silver would insure, if not the total effacement of this contagium, at all events the neutralization of its power to destroy life. The index finger here points out to us the direction which physiological experiment is likely to take in the future. In anticipation of the assaults of infectious organisms, ths experimenter will try to introduce into the body substances which, though small in amount, shall so affect the blood and tissues as to render them unfit for the development of the contagium. And subsequent to the assault of the parasite he will seek to introduce substances which shall effectually stop its multiplication. There are the strongest grounds for the hope that in the case of infectious diseases generally such protective substance will be found.—*New York Med. Times.*

**The Number Seven.**—"Six days shalt thou labor and do all thy work, and on the seventh day rest from thy labors." Probably from the day this command was first uttered there has attached to the number seven something of mystic virtue. In our day there is a very popular belief that "things go by sevens," and the seventh son of a seventh son is believed by a very large proportion of the people, as we find them, to be vested with some power to heal which is not vouchsafed to the general run of mortals, no matter how they may seek to prepare themselves by improving all the advantages of the present "advanced standard of medical education." Hippocrates believed there was "luck in sevens," and he, like Shakespeare, divided the life of man into seven stages, holding that the number seven is the fountain of all the changes in life. For instance, the teeth appear in the seventh month or sooner, and are shed and renewed in the seventh year, when infancy is fully changed into childhood. At twice seven years puberty begins. At three times seven the adolescent faculties are developed, manhood commences, and men become legally competent to complete civil acts At four times seven man is in full possession of all his strength. At five times seven he is fitted for all the business of the world. At six times seven he becomes wise, if ever. At seven times seven he is in his apogee, and from that time decays. At eight times seven he is in his first climacteric. At nine times seven he is in his last or grand climacteric, and at ten times seven he has approached the normal period of life.

There are some remarkable septenary coincidences in the discharge of physiological functions, and in disease processes. The human female menstruates in four times seven days, and in forty times seven days she gives birth to her child. The period of gestation in animals is, in many, if not in all instances, a multiple of seven. In the dog it is nine times seven days; in the cat, eight times seven; in the fox, six times seven. The common hen sits on her eggs three times seven days; the duck and goose four times seven; the crow, three times seven; the swan, six times seven; the peacock, four times seven; the canary and pigeon, twice seven. Bees hatch out in three times seven days. Fever and ague has a tendency to terminate spontaneously after the seventh, fourteenth and twenty-first paroxysms. Relapsing fever is of seven days duration; typhoid fever three times seven days; the incubation of measles twice seven days, and the disease lasts seven days, three of catarrh and four of eruption, before it declines; scarlet fever and erysipelas seven days; smallpox requires twice seven days—from the time of the appearance of the primary fever and the full development of the eruption seven days, and in seven days more the whole crop of pustules has been converted into desiccated scabs.

Truly, there is something wonderful about the number seven.

**Bromidia and Morphia in Delirium Tremens.**—Dr. J. F. Goldman, Huntsville, Ala., writes:

"CASE I.

"Mr. W. R. W., aged thirty-five, a healthy, strong man, had been drinking hard for a number of days, resulting in delirium tremens. I put him on a sol. of morph. and tr. valerian, one ounce each, tr. verat. vir. (Norwood's) one drachm; teaspoonful every hour till sleep. I then went to bed and to sleep, confidently expecting my patient would do likewise; but in this I was doomed to disappointment. Messenger came early in the morning with the information that my patient had been wild all night and slept none. I then prescribed bromidia, two ounces; sulph. morph., two grains; teaspoonful every hour till sleep. The result was most happy. My patient fell into a sound sleep of some twelve hours duration, from which he awakened and went at once to his place of business, a well man.

"CASE II.

"J. S., aged forty, strong, muscular and vigorous. Found him treading the border lands of horrors, with every symptom of delirium tremens. I put him at once on the bromidia aud morph. treatment, with the same result as in case I—sound sleep and perfect recovery. Since treating the above cases, I have relied implicitly on the bromidia and morph. and have never been disappointed."

**Glycerite of Tar.**—The omission of this glycerite from the new Pharmacopœia renders desirable a preparation which may be made without difficulty, and that will enable the pharmacist to furnish the various liquid preparations into which tar enters in a cleanly, easy and expeditious manner. The glycerite made by the following formula, being miscible with water in all proportions, and yielding a clear liquid, commends itself to the favorable consideration of pharmacists:

℞ Oil of tar....................................f ℥ j,

Alcohol.........................................f ℥ ij,

Glycerine and water.............................f ℥ iv,

Magnesia carbonate....................q. s. or f ʒ vi.

Mix the oil of tar with the alcohol, and rub these thoroughly with the magnesia to a smooth paste; to this add the glycerine and water previously mixed together; put the mixture into a well corked bottle and let it remain for several days, shaking it frequently, then filter through paper.

For preparing syrup of tar, add f ℥ ij of glycerite to f ℥ xiv of syrup.

Tar water may be made very readily by using ℥ ss. of glycerite and water sufficient to make a pint.

Wine of tar may be made by using—

℞ Glycerite of tar..................................℥ iij,

Sherry wine.......................................℥ iv,

Syrup.............................................℥ ij,

Water enough to make..............................Oj.

Tar ointment may be made much more easily and much smoother by using two drachms of oil of tar with six drachms of simple cerate.—*American Journal of Pharmacy.—Eclectic Medical Journal.*

**Delirium Tremens.**—Some one has said that delirium tremens is a disease which, so far as its symptomatology is concerned, stands upon a tripod, to wit: sleeplessness, delirium, tremor. I would ask you to make it a four-legged stool, and add the fact, one of the most important of all in differential diagnosis, that there is no fever. Sleeplessness, delirium, tremor, apyrexia; by these signs you shall know it.—*Prof. J. T. Whitaker, Cincinnati Lancet and Clinic.*

**Ergot in Dysmenorrhœa.**—Dr. H. D. McGill (in Southern Practitioner) writes:

"MESSRS. EDITORS: If there is a reader of your valuable journal who has not used ergot in the painful menstruation so common with young females, then he has failed to avail himself of a very valuable remedy. While I am not a very strong believer in specific medicines, yet, if there is such a thing, fl. ext. ergot in one-drop dose, given every hour, has seemed, in my hands, to have a just claim to the title in cases of painful menstruation. (The preparation of Parke, Davis & Co. is the one I have been using.) Two or three doses will generally suffice, and will relieve the distressing headache and backache of the menstrual period, which has produced so many "opium eaters" among the females of the present day. In the same dose, beginning two or three days before the expected catamenial period, it will, in many cases, bring it on, easily and without suffering—proving far more efficacious than preparations of iron or caulophyllin; and I have also found it quite useful in relieving the distressing after-pains so common to many women after parturition.

As a matter of course, there are some practitioners who have used every remedy in every dose that any one else has, according to their statements. They will not be benefitted by my suggestions; but if any one who has never given ergot a trial in the manner I have indicated will do so, I can assure him that he will not be disappointed.

**To Prevent Bed Sores.**—Paint the parts once or twice a day with collodion and castor oil, with equal parts of alcohol and white of egg beaten together, or with "traumaticine" (a solution of gutta-percha in chloroform—4 parts to 30). If an excoriation forms, the following ointment is applied:

℞ Cerate or cold cream..............30 grammes,
Extract of cinchona...........1 to 3 grammes,
Precipitated oxide of zinc..........1 gramme,
Watery extract of opium..........15 centigrammes.
M.

For *phlebitis*, the following application is made along the course of the affected vein:

℞ Purified lard..........................30 grammes,

Watery extract of opium,

Extract of belladonna,

Extract of hyoscyamus,

Extract of conium seed, } aa............3 grammes.

M.

In addition, poultices are used.—*N. Y. Med. Jour.—Amer. Med. Digest.*

**Keeping Cider Without Boiling.**—(Cider, Tyler, Tex.)—Use salicylic acid in the proportion of three grains to each gallon. Sulphite of lime is sometimes used, but we consider the acid to be better suited for the purpose.—*Nat. Drug.*

**Male or Female Offspring at Will.**—What are the laws determining sex? and, Can either sex be produced at will? have always been questions of extreme interest. Dr. Robert Funkhouser recently read a paper before the St. Louis Medical Society (published in the Weekly Medical Review) in which he reports an interesting series of experiments and his conclusions therefrom. He removed the left testicle from a dog, leaving the right intact. This dog could beget male offspring only. The experiment was tried six times with different females, but in every instance the result was male pups only. A dog having only the right testicle and a dog of an entirely different variety were allowed to approach a female at different times. The result was two pups, a male and female. The male was the exact counterpart of the father, having only the right testicle, and the female resembled the dog having both testicles. This was repeated three times, and in each litter there was a male pup resembling the dog having only the right testicle. The reverse of these experiments was tried, removing the right testicle and leaving the left intact, and in every instance female offspring was produced. Similar experiments were made in the female, but it was found that the removal of either ovary made no difference in the sex of the offspring. Females having only the right ovary gave birth to both sexes, and females having only the left ovary gave birth to both sexes, after being approached by dogs having both testicles. So, from this, it seems that the right testicle begets male offspring and the left testicle begets female offspring. The doctor shows that there is greatest affinity between the male and female elements of corresponding sides—that is, there is stronger affinity between an ovum from the right ovary and zoosperms from the right testicle, and vice versa. Then, if an ovum from the right ovary be placed among zoosperms from both testicles, it will become impregnated by the zoosperms from the right testicle by selective affinity. As an ovum is generally, perhaps always, impregnated upon the surface of the ovary or in the fallopian tube, it is plain that if the zoosperms can be gotten in the tube of the desired side, the offspring will be male or female, as the side is right or left. This the doctor does by gravitation. A bitch in heat was impregnated by means of injecting into the uterus seminal fluid collected from a dog. She was then forced to remain lying on the left side for one hour. In due time she gave birth to a female pup. When a male child is desired, the doctor advises that the prospective mother lie on the right side for at least one hour after copulation; if a female child is desired, she should lie on the left side for at least one hour after copulation. The doctor's observations extend over a number of years, and the facts that he has collected and the results of advice given relative to this matter have always been in harmony with the doctrine explained above.—*Med. World.*

**Universal Erysipelas in a Child Aged Ten Months, with Recovery.**—Dr. John Ferguson, of Toronto School of Medicine, has the following in a late number of the American Journal of Obstetrics:

This case is interesting for the following reasons: In the first place, the entire surface of the child became gradually involved, and, in the second place, as illustrating the value of sustaining treatment.

My first visit was made to the little patient on the eighth of May. I was informed that the disease commenced on the day previous, as a reddish swelling on the right labium. When I saw the patient for the first time the vulva was greatly swollen, reddish, slimy, and very tender. The inflammation had also extended on to the pubes and slightly on the lower part of the abdomen.

From this time the disease marched upwards and downwards over the surface of the body, requiring about four days from its appearance at any part until it became nearly normal again in color.

When the erysipelatous inflammation extended to the feet, there was very marked œdema. The chest, back and arms were also taken in turn. The neck and head were next invaded. The eyelids and lips were the last points of attack. In this manner every portion of the entire surface of the body—not omitting the palms of the hands and soles of the feet, which had a tinge of red—was affected at some time during the course of the disease.

The local treatment consisted in frequently and thoroughly anointing the skin with the following:

℞ Acid carbolici........................................gr. v,

Ext. belledonnæ........................................gr. xv,

Ung. petrolei........................................℥ j.

M.

Soft clothes were kept next the skin and child loosely and comfortably covered.

The internal treatment consisted in the administration of one teaspoonful every three hours, in water, of

℞ Quin. sulph........................................gr. iv,
Acid. hydrochlor. dil........................................m. xxxij,
Tr. ferri chloridi........................................fl. ℈ iv,
Syr. simp........................................℥ ij.
M.

As the child was nursing, no other form of food of any kind was given, nor alcoholic stimulants.

The duration of the attack, from its commencement at the vulva to its disappearance at the eyes and mouth, was fifteen days.—*Peoria Med. Monthly.*

**Vomiting of Pregnancy.**—In an obstinate case of this trouble, which had resisted all the usual remedies, the following treatment succeeded: "January 25, at my third visit, I gave her hypodermic injection of mor. sul. gr. ½, sul. atrop, gr. 1-60. In fifteen minutes she was asleep, and slept for several hours—the first good, sound, refreshing sleep she had enjoyed for many days. After she awoke she was able to retain a small quantity of fresh milk with lime water, of which we continued to give her a little every two or three hours. Being a good way from home, and in mid winter, I left the syringe with the husband, with directions how to use it when there were symptoms of the return of vomiting. This was my last visit. He wrote me each day and I sent him directions as to diet, etc. He used the syringe twice after this, each time giving relief. Some two months ago I saw the husband, who reported her doing well, having had but little trouble since my last visit. They have since moved still further away.

Since this I have used a solution of about the same strength, pe. orem, in several cases with good effect, though nct so bad as this case.—*Peoria Med. Monthly.*

**Treatment of Consumption.**—In the Medical Press and Circular, June 8th, Dr. William H. Pearse says he is continually prescribing a combined muriatic acid, quinine aud arsenic treatment to a large number of those who are of the phthisical type, but whose cases have not advanced beyond the indigestion of bodily debility stages, and, with rare exceptions, with marked benefit. His general directions to such a patient are an out-door life, all and any food to which his fancies incline him, and which includes onions and pickles; also cod-liver oil, and the following mixture three times a day after meals:

| | | |
|---|---|---|
| ℞ | Cinchonid. sulph | grs. xx, |
| | Acid muriat. dil | ʒ v, |
| | Liq, arsen. hydro | m. c, |
| | Aq. maris | m. lxxx, |
| | Mangan. sulph | grs. xx, |
| | Infus. quassiæ | ad ℥. |

Twenty doses.

The improvement is steady and regular, though, of course, rather slow.—*New Eng. Med. Month.*

**Cholera Injection.**—Professor Hoven has just published an elaborate lecture in the Revue Scientifique on the treatment of cholera, in which he chiefly advocates ipecacuanha and opium in the earlier stages; and black sulphide of mercury with salicylate of bismuth in the more advanced periods. When the patient falls into the state of algidity and collapse, the professor recommends the intravenous injection of the following solution (Medical Times):

| | | |
|---|---|---|
| ℞ | Aq. simpl | 1000 parts, |
| | Chloride of sodium | 5 " |
| | Hydrate of sodium | 1 " |
| | Sulphate of sodium | 25 " |

—*Louisville Medical News.*

**Cocoa.**—Squibbs' *Ephemeris* says that the difficulty in obtaining a good article of cocoa in the markets has become so great as to cause the writer to abandon the manufacture of the fluid extract, and to substitute for it an extract of tea (Thea Viridis). Cocoa, according to the same authority, has not commended itself generally to therapeutic use.—*Pacific Medical Journal.*

**Copper as an Antidote to Cholera.**—At a recent meeting of the Academie des Sciences, M. Bouley drew attention to M. Burk's assertion that those persons whose organisms are thoroughly submitted to the influence of copper are as inaccessible to the attacks of cholera as those vaccinated are to smallpox. The following methods, according to M. Burk, are all equally efficacious: the habit of wearing copper bracelets, or bands which encircle the waist, or materials which have been steeped in solution of copper, or the administration of black oxide of copper in the form of pills. At a recent meeting of the Academy of Medicine, M. Bailly furnished personal evidence which invalidated M. Burk's statement concerning the therapeutic value of copper in treating cholera. M. Bailly practices at Chambly, very near manufactories where spoons and forks are made with a copper alloy known as alfenide. All hands employed exhibit symptoms of the influence of copper; nevertheless, the ravages made in 1866 by an epidemic of cholera are subversive of M. Burk's hypothesis. During an epidemic of typhoid fever, fifty-six people were attacked; of these, twenty-six were impregnated with copper. Four of the twenty-six died; no other deaths are recorded. M. Bailly mentioned a fatal case of "charbon" consequent on a fly-sting; also, deaths from diphtheria. All the sufferers exhibited symptoms of copper-impregnation. Summer diarrhœa, also choleraic diarrhœa, attack those among the work-people who are thoroughly impregnated.—*British Medical Journal.*

**Deep Breathing.**—The composition of nitrous oxide (laughing gas) is the same as that of common air, except that it has a larger proportion of oxygen, and its anæsthetic effect is thought to be due to its oxygen. Deep and rapid breathing, by supplying an excess of oxygen to the blood, has been found to be an anæsthetic of considerable value. Many operations rendered painless by this measure have been reported. We wish to suggest that this forcible injetcion of oxygen (oxygen is a gaseous food) may be made a valueable therapeutic procedure. Habitual deep breathing will doubtless benefit many cases of anæmia, mal-nutrition, etc., provided that the air be pure. Indeed, we can scarcely imagine any abnormal condition that might not be benefitted in this way. Those persons who lie awake at night and toss about restlessly, vainly trying to go to sleep, will find deep and rapid breathing for several minutes to be a sweet and grateful composer.—*Med. World.*

**Post-Nasal Catarrh.**—Dr. Curtiss, of St. Louis, thus cures Naso-Pharyngeal Catarrh (St. Louis Med. Jour.): The treatment is conducted by means of sprays, and the fluids used are composed as follows: A cleansing fluid is used, first, of boracic acid, glycerine and water; after the cleansing process, a spray of melted cosmoline with a proportion of five grains in two ounces of corrosive sublimate. The "cure" in this is very prompt: taking about ten treatments, the discharge stops and the mucus membrane resumes its natural appearance. He mentions this as a type of no less than twenty cases which he has treated successfully within a year past.

**Conium in Malarial Disease.**—Richard C. Newton states that he has frequently used the following prescription in the treatment of malarial disease:

℞ Ext. conii fl.................................ʒ x,

Ferri peroxid.................................ʒ iij,

Sp'ts vin. gal................................℥ iss,

Quin. sulph.................................ʒ ss,

Syr. simp.................................℥ iij,

Ol. menth. pip.................................ʒ iij.

M. Sig. ʒ j every four hours; then every two hours for two or three doses; then every four hours.

To children it has been given very successfully by doubling the amount of syrup.

He says the prescription was given to him by Mr. F. O. Vaille, of Denver, Col., who had it from his father, the late Dr. Vaille, of Springfield, Mass. The remedy has a high degree of local popularity as "Vaille's medicine." Dr. Newton finds it a "mild laxative, diaphoretic and tonic. Its quieting and soothing effects differ widely from the disturbance often occasioned by quinine." He regards it as a safer remedy than Warburg's tincture.—*Medical Record.*

**The Bromides as a Cause of Insanity.**—At the last meeting of the American Neurological Association, Dr. Bannister called attention to the maniacal excitement sometimes produced by the use of the bromides. Corroborative instances were mentioned by Drs. Hammond, Jewell, Spitzka and Seguin. Perhaps the best commentary on this paper is the recent suicide of a physician in a condition of frenzy produced by bromidizing himself for the prevention of sea-sickness. This procedure has been so enthusiastically recommended for sea-sickness, by the author of "Oh, My," Dr. Beard, that it is about time some attention was called to the dangers of the process. The bromides have been greatly abused in the treatment of the neuroses, and it is to be hoped that a severely critical examination of their therapeutic use will be inaugurated by this paper of Dr. Bannister.—*Chicago Med. Rev.*

**Witch Hazel in Menorrhagia.**—The Medical Record quotes from an article by Dr. Henry M. Chute, in the South African Medical Journal, in which he reports the results of his use of hamamelis virginica in the treatment of the menorrhagia, which is a very frequent ailment of women in Cape Colony. He administered the drug in doses of half a teaspoonful of the fluid extract in sugared water, two or three times a day. He says it acts so quickly that it is not necessary to anticipate the flow, and when the menstruation has lasted the ordinary time and is not closing naturally the remedy will effectually restrain it; but after the hemorrhage has ceased there is no advantage in continuing it. Another good result of the drug is observed in dysmenorrhœa, in which it, in a very marked, quick manner, relieves the pain.—*Ther. Gaz.*

**A New Disease.**—Our British colleagues announce what they claim to be a new disease, which manifests itself by excavating ulcers, with an irregular caseous-looking base, a semi-gelatinous centre, and affects the region of the chest, neck and jaw.

The Pathological Society sets it down as a new infectious disease, to be known as actino-mycosis, that its origin was with cattle, and formerly known as maxillary sarcoma.

Prof. Ponfick, of Breslau, was first to describe the disease in man as identical with that found in cattle, in his monograph entitled, "Human Actino-mycosis." It is said that the growths are distinctly pedunculated, and are fungoid in character.—*New York Med. Times.*

**Treatment of Ingrowing Toe Nail.**—Professor Petersen removes the whole of the soft part down one side of the nail, extirpates the nail itself, from antiseptic motives, and, after arresting bleeding by pressure, dresses the wound rapidly with oxide of zinc and cotton-wool. Fourteen days rest in bed, with the foot raised, generally suffices for cure, and the contraction of the cicatrix prevents a relapse to the old condition. Professor Petersen prefers general to local anæsthesia, on account of the troublesome bleeding afterward; and for the same reason he does not approve of Esmarch's bandage in this operation.—*Cin. Lancet and Clinic.* [This is *toe* many operations.—EDITOR RECORD.]

**The Globus Hystericus.**—Dr. Roth regards this symptom as due to a paræsthesia of the sympathetic. And as the pellitory root has been found useful in paralysis of the tongue and pharynx, the author was led to try it in globus. He gives from ten to twenty drops of the tincture of pyrethrum four times a day. He reports six cases in which he employed this remedy with satisfactory results.—*Centralblatt fur Gynakologie, May 3, 1884.*

**Cure of Hydrocele.**—A correspondent asks for Levis' plan for the radical cure of hydrocele with carbolic acid. Ans.: Tap the hydrocele and inject from one to two drachms of 95 per cent. carbolic acid into the sack. If the fluid should again accumulate, remove it by tapping. It is then said not to return.—*Medical Review.*

**Cholera Preventives.**—Cholera germs require an alkaline medium for their development. For this reason acids should be used freely by those exposed to the poison. The mineral acids, preferably the aromatic sulphuric acid, have proved to be the most efficient prophylactics ever tried, and their value should be more generally acknowledged.—*Med. World.*

In the Detroit Lancet, December, 1883, Dr. A. B. Ballou reports a case of complete fracture of the upper portion of the left side of the forehead and escape of brain substance, with perfect recovery.

